# Pharmacy of the Greeks and Romans

**Published:** 1851-09

**Authors:** 


Art. III.—*Pharmacy of the Greeks and Romans.* Translated from the French of M. Cass; by Wm. H. Cobb, M.D., of Louisville, Kentucky.

Claudius Galen was born at Pergamus, in Asia Minor, in the 131st year of the Christian era, and died in the 201st year, at the age of 70. He lived, consequently, under the emperors Adrien, Antoninus, Marcus Aurelius, Lucius Verus, Commodus, Pertinax, and Septinus Severus. His father, named Nicon, was an architect and senator. A dream determined him to choose the study of medicine for his son. He was rich and learned, and devoted himself assiduously to the education of young Galen. The latter, after the death of his father, and when he had arrived at the age of 21, betook himself to Smyrna, to study philosophy, then to Corinth, and afterwards to Lycia and Palestine, to investigate the jet and asphaltus. Alexandria was then the center, as it were, of literature. There Galen passed many years, and perfected himself in the study of anatomy, one of the branches of the science which he made the favorite object of his researches.

At the age of 28 years, he returned to his own country, where the priests of Æsculapius entrusted him with the office of attending upon the athletes. Some years after, a revolution having arisen in Pergamus, he quitted that city and repaired to Rome; he was then 34 years old. There he dislocated his arm, which, however, did not hinder him from attaining great celebrity. He allied himself with the savants, the philosophers, and great personages, and among others with Severus, who afterwards became emperor. The following year, during an epidemic which raged at Rome, he went to Brindes and embarked for Greece. He visited Cyprus, and brought thence the diphrix or diphriges (the oxide of copper) then much employed as an astringent and detergent. His voyages, always undertaken for the purpose of increasing his knowledge, proved to him so many opportunities for making useful researches in natural history. Thus at Cyprus, he studied metallurgy; at Palestine he investigated the opobalsamum; at Lemnos the terra sigillata. He was 38 years of age when Marcus Aurelius and Lucius Verus, who were then in Illyria to carry on the war against the Macedonians, summoned him before them. He traversed, on foot, Thrace and Macedonia, and appeared before them at Alexandria; there he prepared the Theriaca Andromachi, for the use of the two emperors. The plague having broken out and destroyed Lucius Verus, Galen returned to Rome, where he was appointed physician to the youthful emperor, Commodus. He was more than 50 years old when he returned to his own country, where he died, says, Suidas, at a very advanced age.

Without entering minutely into the medical doctrines of Galen, still we cannot give an idea of his system, relative to medicines, without touching upon certain general matters appertaining to these doctrines; this we do, nevertheless, with all the reserve which our principal object imposes. Here then, in a few words, is this system:—

The elements of all bodies, according to the theory of Empedocles, adopted by Hippocrates, are fire, water, air, and earth. The qualities of these elements are, heat and cold, dryness and moisture. These same qualities are met with in medicines, and each part of our organism exercises upon them an action which depends upon the similitude existing between the organ and the medi ine.

As long as no one of these elements predominates in the organism, and the parts of which it is composed, have the proper blending temperament among themselves, they perform their functions in a regular manner. But as soon as one of these is in excess, the equilibrium is destroyed, it results in loss of the proper blending; and the functions are altered. That these organic constituents may vary in their size, figure, number, and situation, it even suffices that they merely depart from their ordinary condition; this it is, which constitutes good or bad health.

Medicine has two objects, the preservation of health and the cure of disease. The duty of the physician is to maintain th *temperaments* and to correct any departure from it, by remedies bearing a relation to these different conditions.

The kind or cause of disease always indicates the remedy, but there are causes which exist in the nature of the organism itself, and others which result from accidents. What nature has not accomplished it is clear the physician cannot accomplish, but he can second her efforts for re-establishing the equilibrium necessary to the state of health.

The elementary qualities predominating in an individual, constitute what is termed his temperament. These qualities may be combined among themselves, multiplying the varieties of temperament.

Every active medicinal substance, like the elements of nature, can be hot or cold, dry or humid. To the knowledge of these primary qualities we attain, by that of their secondary qualities, the physical properties. The physical properties of medicine consequently determine the manner of using them.

The primary qualities have various degrees. The chicory, for example, is cold in the first degree, pepper hot in the fourth. The taste and other sensible properties, as bitterness, acidity, and saltness, belong to the primary qualities. Saltness, for example, is warm, bitterness is dry, and acidity is cold, &c. These qualities may belong to substances either directly or indirectly. Ice is of itself cold, mandragora and the hemlock are cold merely by the effects they produce. Galen divides medicinal agents, relatively to their action, into specifics, poisons, and counter poisons. Purgatives, according to him, act by the whole of their substance, and each possesses the power of attracting a particular humor corresponding to its own nature.

But let us not enter more deeply into this exposition. Does it not surpass belief that such a system should be the most brilliant result of the meditations of antiquity relative to the action of medicines? Can we conceive it possible that this doctrine could be maintained during the 13 centuries it reigned in Europe, Africa, and Asia, and that it should flourish also during our own times among certain nations, but little advanced in science, it is true, as the Turks and Arabs? This is nevertheless the truth, and it is one of the most striking examples of the authority which, even in the sciences, the talent for exposition, the prestige of the name, and the personal merit of a superior man can exercise. To explain such wonderful success, we must seek for the cause in the extraordinary events attending the appearance of the philosopher of Pergamus.

Medicine was then divided into a multitude of sects and schools, in which reigned vain subtilties and futile discussions. Galen did not wish to belong to any one of these schools, but sought to lead science into the path of Hippocrates, the path of truth. He collected the doctrines and opinions of all the celebrated men who had preceded him, and from this rubbish he raised a system ingenious and easy of comprehension, which he developed by the aid of instruction full of charms and seductive graces. He put an end to the uncertainties of his time, and devoted himself to introducing into medicine the scientific forms borrowed from the peripatetic school. Talents of an elevated order, unquestionable genius, the number of his writings, the order and logic whieh characterize them, the purity and elegance of his style, all, even his egotism and assurance, contributed to captivate the imagination and to earn for him a glory which for its eclat and duration in the history of the sciences, can only be compared with that of Aristotle.

Galen produced many excellent philosophical works. In his youth, he had written commentaries upon the logic propounded by Chrysippus; in them, he expressed himself with great facility—his knowledge is universal. The number of his writings amounts to not less than five hundred. In our day, we do not possess more than a third of them. Among these writings are found many upon medicinal substances, and a much larger number upon the composition of medicines. The fourth and fifth part of his works contain eleven books which treat of simple medicines, one of succedanea or quipropro, two of the theriaca and mithridate, seven of the composition of medicines by genera, and ten of the composition of medicines according to the places of their application.

Not content with having collected all that his predecessors had written upon this subject, Galen got together from all quarters receipts of pharmaceutical preparations, and oftentimes purchased them at a very high price. Although he approved of the simplicity and reserve of Hippocrates relative to the employment of medicinal agents, yet it did not prevent him from making use of them in their most complicated forms and in great number. Materia medica is, in fact, one of the points upon which he differed most widely from the physicians of Cos, and yet the most monstrous of these masses of drugs cannot with justice be attributed to him. We may even remark that his own formulas are less loaded with them than those borrowed from others. He condemns, in more than one instance, the abuses which are practiced in them; he blames physicians who recommend cosmetics, and especially those who taught the preparation of poisons; but the care which he took to write so lengthily upon pharmacology sufficiently evinces the importance he attached to this subject. We may add that his example has exerted much influence upon the combination of medicines, a combination in which the Arabs especially excelled and which has been handed down almost to our day. It is because he is the physician who has written more at length on this subject that this division of the art has borne for so long a time the name of Galenic pharmacy. The distinction between the Galenic and chemical pharmacy was established about the time of Paracelsus and Van Helmont, for the purpose of separating the physicians who still cling to the doctrine of Galen from those who rallied under the banners of the new sect. We know that Paracelsus strove to substitute for the system of the elementary qualities that of the chemical elements recognised by his own school, a system which was linked by more than one tie to the absurdities of the cabala, and which so long retarded the upward flights of rational chemistry. In the majority of the pharmacopeias of the seventeenth and even of the eighteenth century, medicines are divided into two great classes, the first comprehends all medicines of the organic kingdom prepared by simple mixture, and in which we do not suspect the occurrence of chemical decomposition; the second includes all those into whose composition enter acids, alkalies, salts, metals, &c., and in which chemical phenomena play an important part.

The authority of Galen with respect to medicines continued long after the authority of his medical doctrines had ceased to exist. During the sixteenth century, the former commenced to diminish, and early in the seventeenth century the discovery of Harvey gave it a final blow. Like Aristotle, after having for thirteen centuries been the oracle of science, mankind ended by denying him even those titles of glory to which he was most justly entitled. But if the respect and enthusiasm which he inspired during so long a period was exaggerated, the neglect and ingratitude of moderns is not less so.—Galen is the last and one of the most eminent physicians of whom antiquity can boast. No one was ever endowed with a genius more brilliant, erudition more profound, or talents more varied. To him we are indebted for the preservation of the precious remains of antiquity and of the true principles of the school of Cos. His authority in medicine equalled that of Aristotle in philosophy. His writings are the most abundant and the best arranged that the science of ancient times has handed down to us. Are we then to blame him for being the object of a superstitious idolatry at an epoch when medicine derived nothing from its own ranks, but subsisted merely upon that which was bequeathed to it by one of the most brilliant periods of civilization? A mind of the most refined order, the purest taste, adorned this genius, already so powerful by its knowledge, the most extensive, perhaps, of his time. His elocution was rich and facile, his style so pure and elegant as to remain as a classical authority. Perhaps he sometimes abused these rare qualities and presumed upon the influence which they lent him. The desire of shining forth, of rendering truth more captivating, of explaining even that which was inexplicable, oftentimes bore him beyond his proper limits. But ought we not to pardon something in superior minds, and whatever may be the height attained by their talents, do they not cling by a certain feebleness to the conditions of human nature?

Galen was superstitious; he believed in dreams and divinations. He had a veritable worship for Æsculapius, whom he styled the father of his country, and who, he believed, inspired him with the employment of certain curative agents. He says somewhere in his works that the sedative properties of the juice of the lettuce were revealed to him by the dream of one of his patients. His vanity was excessive. Hippocrates indeed opened the way to medical research, said he, but it was he himself who leveled the opposing difficulties, as Trajan rendered practicable the routes of the Roman empire. Like the greater part of the physicians of his period, Galen practiced at the same time the different branches of the healing art. He especially practiced pharmacy, and occupied, as he himself says, an office in the *Via Sacra*. This office was destroyed by a conflagration under the reign of Commodus. A part of his works was consumed by another conflagration which consumed the temple of Peace. Is it then a paltry glory—are these vain titles to renown for a scientific profession to be able to cite among those who have practiced it such men as Hippocrates, Aristotle, Theophrastus, and Galen—of combining among the number of those who have served in its ranks or celebrated its praises, heroes, monarchs, and poets?

Thus it was that the pharmacy of ancient times was celebrated. In scanning the history of modern times, we shall not fail to find new claims to the esteem of the learned and to the gratitude of humanity.

